# A review on functional and structural brain connectivity in numerical cognition

**DOI:** 10.3389/fnhum.2015.00227

**Published:** 2015-05-13

**Authors:** Korbinian Moeller, Klaus Willmes, Elise Klein

**Affiliations:** ^1^Knowledge Media Research CenterTübingen, Germany; ^2^Department of Psychology, Eberhard-Karls UniversityTübingen, Germany; ^3^Department of Neurology, Section Neuropsychology, University Hospital, RWTH Aachen UniversityAachen, Germany

**Keywords:** brain connectivity, DTI, white matter pathways, fronto-parietal network, numerical cognition

## Abstract

Only recently has the complex anatomo-functional system underlying numerical cognition become accessible to evaluation in the living brain. We identified 27 studies investigating brain connectivity in numerical cognition. Despite considerable heterogeneity regarding methodological approaches, populations investigated, and assessment procedures implemented, the results provided largely converging evidence regarding the underlying brain connectivity involved in numerical cognition. Analyses of both functional/effective as well as structural connectivity have consistently corroborated the assumption that numerical cognition is subserved by a fronto-parietal network including (intra)parietal as well as (pre)frontal cortex sites. Evaluation of structural connectivity has indicated the involvement of fronto-parietal association fibers encompassing the superior longitudinal fasciculus dorsally and the external capsule/extreme capsule system ventrally. Additionally, commissural fibers seem to connect the bilateral intraparietal sulci when number magnitude information is processed. Finally, the identification of projection fibers such as the superior corona radiata indicates connections between cortex and basal ganglia as well as the thalamus in numerical cognition. Studies on functional/effective connectivity further indicated a specific role of the hippocampus. These specifications of brain connectivity augment the triple-code model of number processing and calculation with respect to how gray matter areas associated with specific number-related representations may work together.

In the history of neurology, attempts to explain normal and impaired cognitive function following brain damage have alternated between two extreme perspectives; specifically, views based on localization of function and views based on functional connectivity. The localizationist view ascribes specific cognitive functions to gray matter (GM) brain areas with cognitive impairments attributed to lesions of these specific areas. Prominent historical examples of this view include the work of Broca (1861) and Wernicke (1874), who associated language production and perception, respectively, with specific cortical structures. Another prominent example of localization of function is the work of Brodmann (1909), who proposed a map of 46 cortical areas—so-called Brodmann areas (BA)—and their functionality. This work still influences neuro-scientific research today. In contrast, connectionist views of brain function take the connections of white matter (WM) pathways to be instrumental to cognitive functions, with disrupted connections also leading to impairments of the respective cognitive functions. Interestingly, such a connectionist view of brain function was proposed by Campbell (1905) at about the same time as Brodmann introduced his localizationist approach. Later, Reinvang (1985), amongst others, suggested “systemic localization” to be the overarching principle of brain organization, in which the functional role of a given brain area is not determined by its anatomical structure alone but also by its relationships to other areas—an argument, for which there is increasing empirical evidence (e.g., López-Barroso et al., 2013; see Catani et al., 2012, 2013 for reviews). Thus, it is the integrity and specific interplay of activated GM cortical areas connected by WM fiber tracts which underlie human cognitive functions.

Recently, brain hodology, the science of connectional anatomy (Catani and Ffytche, 2005), which characterizes the WM connections between brain regions, has become accessible to evaluation in the living brain by using diffusion tensor imaging (DTI). While functional magnetic resonance imaging (fMRI) identifies functionally defined cortical areas, tractography goes beyond this approach and indicates, by which WM tracts these areas are connected. This provides a powerful tool to study brain connectivity patterns underlying cognitive functions. By quantifying the diffusion characteristics of water molecules (Le Bihan and Breton, 1985), which diffuse more freely along than across myelinated tracts, it is possible to obtain *in vivo* estimates of WM fiber orientation at the voxel level (Basser et al., 1994). This information gives rise to diffusion tensor tractography (Conturo et al., 1999; Jones et al., 1999; Mori et al., 1999; Basser et al., 2000; Poupon et al., 2000), in which WM tracts are reconstructed in three dimensions by sequentially piecing together discrete voxel level estimates of fiber orientation to extrapolate continuous trajectories. Diffusion tensor tractography methodology has established the existence of neural networks associated with language processing (e.g., Saur et al., 2008) and also networks subserving attentional functions (e.g., Umarova et al., 2010). Accumulating such evidence has substantiated the functional role of WM connections in both language as well as attentional processing (e.g., Rijntjes et al., 2012). There have even been suggestions to conceptualize aphasia (e.g., Forkel et al., 2014) and neglect as disconnection syndromes (e.g., Bartolomeo et al., 2012; Thiebaut de Schotten et al., 2014) arising from disrupted neural connections between the involved cortex areas.

Numerical cognition and the syndrome of acalculia, a collection of impairments in processing numbers and mental calculation, have also witnessed a history of localisationist and connectionist views, although their study started later in history and they were less well investigated than language. Henschen (1920), who coined the term acalculia, also considered calculation mechanisms to rely on a complex anatomo-functional system, subserved by distinct cortical centers and their interconnections.

In the present paper we summarize and review the existing evidence on brain hodology underlying numerical cognition. Comparable to the cases of language and attention, considering WM connections may provide a more comprehensive understanding of human numerical cognition and its impairments (see also Matejko, 2014; Matejko and Ansari, 2015). First attempts were made to conceptualize acquired acalculia (Klein et al., 2013b) but also its developmental counterpart dyscalculia (DD) as disconnection syndromes (Kucian et al., 2014). Therefore, we will first give a brief overview regarding the neural GM correlates of numerical cognition before augmenting those neuro-functional data with recent evidence on WM connectivity made accessible by technical advances in DTI. In this review we use a broad definition of numerical cognition that encompasses tasks reflecting basic numerical competencies (e.g., magnitude comparison) but also mental arithmetic (e.g., addition, subtraction, multiplication, etc.), as also required in standardized tests of mathematical and/or intellectual abilities. Studies investigating higher mathematics (such as algebra, analysis or inferential procedures, etc.) and their neuro-structural correlates are not included in the current review.

## Neural Correlates of Numerical Cognition

In the past two decades, significant progress has been made in uncovering the neural basis of numerical cognition (Menon, in press, for a review). The triple-code model (TCM) of Dehaene et al. (2003) reflects a unique integration of behavioral and neuro-functional aspects, proposing three different representational codes for numbers and their neural correlates. (i) A bi-hemispheric *numerical magnitude representation* associated with the intraparietal sulcus (IPS); (ii) A *verbal representation* of numbers associated with left perisylvian language areas and the left angular gyrus (AG) which is recruited in verbally mediated operations like number naming as well as arithmetic fact retrieval; and (iii) A *visual number form* *representation* specialized for recognizing Arabic digits and associated with bilateral fusiform regions. From its initial form the TCM assumed that number processing requires the close interplay of domain-specific number-related parietal as well as domain-general (pre)frontal processes involving working memory and executive control. This suggests that numerical cognition is subserved by a multi-modular and distributed system within the human brain.

So far, the TCM has not taken into account an explicit and detailed delineation of the connecting fiber pathways subserving this multi-modular organization, probably due to the non-availability of appropriate imaging methods at the time of its initial formulation. Nevertheless, in the first version of the anatomo-functional TCM (Dehaene and Cohen, 1995), and in a series of subsequent detailed single case studies, the involvement of intra-hemispheric (cortico-subcortical, fronto-parietal) as well as inter-hemispheric (commissural) pathways for number processing and calculation was highlighted. Moreover, observed patterns of impairment (e.g., pure alexia for numbers) were also explained by a disconnection account (Cohen and Dehaene, 1995; see also Klein et al., 2013a). Nevertheless, the vast majority of recent neuroimaging studies have focused on the localization of activated GM areas. WM connections underlying numerical cognition were not considered specifically in most cases. We identified 10 studies investigating functional connectivity (Table 1), and 17 studies investigating structural WM connections in numerical cognition (see Table 2) from the last ten years. The increasing number of publications in recent years may not only reflect increasing research interest but also progressive availability and validity of DTI sequences (e.g., Soman et al., 2015) and appropriate processing software.

**Table 1:** **Overview of studies investigating functional/effective connectivity underlying numerical cognition**.

Nr.	Authors	Year	Connectivity analysis	Task	Participants	Connections
**1**	Tang et al.	2006	Functional connectivity	Magnitude comparison, addition	Chinese: 23.8 ± 0.8 years; English-speaking: 26.8 ± 2.3 years	VFG – SMA, L SMA – L PMA, L PMA– Broca, Broca – Wernicke, VFG –L IPC, L IPC – Wernicke
**2**	Krueger et al.	2011	Effective connectivity (GCM)	Multiplication	26 ± 6.7 years	R IPS – L IPS; R IPS – R DLPFC; L precG,- L preSMA; L preSMA –L/R DLPFC; L IPS –L DLFPC
**3**	Rosenberg-Lee et al.	2011	Functional connectivity	WIAT, WMTB-C	7–9 years	L DLPFC –L AG, L SPL
**4**	Cho et al.	2012	Effective connectivity (PPI)	Addition	7–10 years	R Hippocampus –L DLPFC; L VLPFC
**5**	Emerson and Cantlon	2012	Functional connectivity	TEMA, Matching numbers, faces, words, and shapes	4–11 years	IPS –PFC, IFG, insula
**6**	Supekar et al.	2013	Functional connectivity	WASI; WIAT, WMTB-C, Reading, addition verification and production	8–9 years	R Hippocampus –R MTG, R SMA, L DLPFC, L VLPFC, L BG
**7**	Park et al.	2013	Effective connectivity (PPI)	non-symbolic Addition, number matching, shape matching	18–29 years	R IPS –L IPS, L sensorimotor cortex
**8**	Park et al.	2014	Effective connectivity (PPI)	Magnitude comparison on digits, dots, and line lengths	4–6 years	R SPL –L SMG, R preCG
**9**	Qin et al.	2014	Effective connectivity (PPI)	Addition	7–9, 14–17, & 19–22 years	Hippocamus –L/R DLPFC, L IPS
**10**	Rosenberg-Lee et al.	2015	Effective connectivity (PPI)	Addition, subtraction	7–9 years, 16 with dyscalculia	Hyperconnectivity IPS –AG, L SMG, R MFG, R IFG, VMPFC in dyscalculia

*L: left; R: right; GCM: Granger causality mapping; PPI—Psycho-Physiological Interactions; TEMA: Test of Early Mathematics Ability (Ginsburg and Baroody, 2003); WMTB-C: Working Memory Test Battery for Children (Pickering and Gathercole, 2001); WASI: Wechsler Abbreviated Scale of Intelligence (Wechsler, 1999); WIAT: Wechsler Individual Achievement Test (Wechsler, 2005); VFG: visual fusiform gyrus; PMA: premotor association areas; Broca: Broca’s area; Wernicke: Wernicke’s area; IPC: intraparietal cortex; IPS: intraparietal sulcus; DLPFC: dorsolateral prefrontal cortex; (pre)SMA: (pre) supplementary motor area; preCG: pre central gyrus; SPL: superior parietal lobe; AG: angular gyrus; VLPFC: ventrolateral prefrontal cortex; IFG: inferior frontal gyrus; MTG: middle temporal gyrus; BG: basal ganglia; SMG: supramarginal gyrus; VMPFC: ventromedial prefrontal cortex*.

**Table 2:** **Overview of studies investigating structural connectivity underlying numerical cognition**.

Nr.	Authors	Year	Connectivity analysis	Task	Participants	White matter tracts
**1**	Barnea-Goraly et al.	2005a	DTI, ROI analysis (6 directions)	WISC number tasks	7–20 years, VCFS	–
**2**	van Eimeren et al.	2008	DTI, ROI analysis (32 directions)	WIAT number tasks	7–9 years	Atlas-based: SCR, ILF
**3**	Rykhlevskaia et al.	2009	DTI, fiber tractography (probabilistic and deterministic, ROI analyses), 23 directions	WASI, WIAT, WMTB-C	7–9 years, 23 with dyscalculia	Tractography-based: ILF, IFOF, thalamic radiation, caudal forceps major
**4**	Tsang et al.	2009	DTI, ROI analysis (12 directions)	Multiplication, exact and approximate addition, WISC, WRAT, Reading	10–15 years	Atlas-based: SLF
**5**	van Eimeren et al.	2010	DTI, ROI analysis (12 directions)	Four basic arithmetic operations	26.4 ± 3.0 years	Atlas-based: SCR
**6**	Cantlon et al.	2011	DTI, fiber tractography ROI analysis (deterministic, 15 directions)	Number comparison symbolic and non-symbolic	6 years	Tractography-based: Callosal isthmus
**7**	Hu et al.	2011	DTI, TBSS analysis (15 directions)	Digit/letter span, WAIS, 3 years of abacus training	10 years	Atlas-based: Internal capsule, thalamic radiation, corona radiata, SLF, ILF
**8**	Klein et al.	2013b	DTI, fiber tractography ROI analyses (probabilistic, 61 directions)	Mental addition	28 ± 5 years	Tractography-based: SLF, EC/EmC
**9**	Klein et al.	2013a	Fiber tractography (deterministic)	–	49 years, single case	Tractography-based: EC, SLF
**10**	Kucian et al.	2013	DTI, ROI analysis (21 directions)	ZAREKI, WISC, Corsi	10 years, 15 with dyscalculia	Atlas-based: SLF, adjacent to IPS
**11**	Navas-Sanchez et al.	2013	DTI, ROI analysis (16 directions)	Math Talent Program, Madrid, Spain	12–15 years	Atlas-based: Corpus callosum, internal capsule, SLF, SCR, EC, thalamic radiation
**12**	Matejko, et al.	2013	DTI, TBSS analysis (31 directions)	PSAT	17–18 years	Atlas-based: SLF, SCR, corticospinal tract
**13**	Li et al.	2013a	DTI, fiber tractography (probabilistic and TBSS, 30 directions)	WISC	10–11 years	Tractography-based: SLF, ILF, inferior fronto-occipital fasciculus
**14**	Li et al.	2013b	DTI, fiber tractography (probabilistic and TBSS, 15 directions)	Abacus training for 3 years	10 years	Tractography-based: Forceps major
**15**	Willmes et al.	2014	DTI, fiber tractography, ROI analysis (deterministic, 61 directions)	Parity judgment, magnitude comparison from Klein et al. (2010)	18–25 years	Tractography-based: EC/EmC, SLF
**16**	Van Beek et al.	2014	DTI (45 directions)	Addition, Subtraction; Multiplication, Division, WISC, WMTB-C, word and pseudoword reading	11–13 years	Anterior arcuate fasciculus
**17**	Klein et al.	2014	DTI, fiber tractography (deterministic, 61 directions)	Number bisection, exact/approximate addition	19–42 years	Tractography-based: MdLF, ILF, SLF, EC/EmC, cingulate bundle

*PSAT: Preliminary Scholastic Aptitude Test (College Board USA, 2006); WASI: Wechsler Abbreviated Scale of Intelligence (Wechsler, 1999); WIAT: Wechsler Individual Achievement Test (Wechsler, 2005); WISC: Wechsler Intelligence Scale for Children (Wechsler, 2004); WMTB-C: Working Memory Test Battery for Children (Pickering and Gathercole, 2001; WRAT: Wide Range Achievement Test (Wilkinson and Robertson, 2006); ZAREKI-R: Testverfahren zur Dyskalkulie bei Kindern (von Aster et al., 2005)*.

Almost all studies aimed at specifying the fronto-parietal network underlying numerical cognition as suggested by the TCM. In this vein, intra-hemispheric fronto-parietal connections (e.g., Rykhlevskaia et al., 2009; Tsang et al., 2009; Matejko et al., 2013; Navas-Sánchez et al., 2014) and inter-hemispheric (intra)parietal to (intra)parietal connections (e.g., Cantlon et al., 2011; Krueger et al., 2011; Klein et al., 2013b; Park et al., 2013) were of primary interest in most studies. In the following we will summarize and review the existing evidence regarding brain connectivity in numerical cognition. First, functional and effective connectivity (reflecting correlations between activation in specific brain areas) will be considered. Subsequently, we will elaborate on studies addressing structural connectivity, which allow identification of anatomical WM fiber tracts involved in numerical cognition.

## Brain Connectivity in Numerical Cognition

### Correlations Between Activated Brain Areas—Functional and Effective Connectivity

A first way of evaluating the connectivity between specific brain regions involves computing functional connectivity; specifically, the correlation patterns between neural *GM* activation elicited in different brain regions, while performing a specific numerical task. Highly correlated activation in two different brain areas is assumed to indicate that these areas may work together (see Table 1 for an overview of studies investigating functional connectivity). Emerson and Cantlon (2012) used a symbolic-nonsymbolic number matching task to localize number-specific activation in parietal and (pre)frontal cortex areas in four- to eleven-year-old children. They then correlated the time series of activated voxels within frontal regions of interest (ROIs) with parietal ROIs to obtain a measure of fronto-parietal connectivity. Interestingly, stronger fronto-parietal connectivity was associated with better math proficiency, emphasizing the importance of integrated fronto-parietal processing in numerical cognition. Tang et al. (2006) observed differential patterns of fronto-parietal functional connectivity for Chinese- and English-speaking participants in both a magnitude comparison task and a mental addition task. The authors argued that Chinese-speaking participants seemed to engage more strongly a visuo-premotor association network for solving these tasks (involving visual fusiform gyrus and premotor association areas). On the other hand, native English speakers seemed to largely employ language-based processes relying on left perisylvian cortices (including Broca’s and Wernickes area) for the same tasks.

The important role of integrated fronto-parietal processing was further substantiated by Supekar et al. (2013), who investigated the neural predictors of arithmetic skill acquisition in 8–9-year-old children before an 8-week math tutoring program. The authors found that functional connectivity of the hippocampus with dorsolateral and ventrolateral prefrontal cortices as well as with the basal ganglia prior to tutoring predicted subsequent learning effects. This finding was interpreted to indicate that “individual differences in the connectivity of brain regions associated with learning and memory, and not regions typically involved in arithmetic processing, are strong predictors of responsiveness to math tutoring in children” (Supekar et al., 2013, p. 8230). In another study evaluating the manifestation of numerical learning in brain connectivity Rosenberg-Lee et al. (2011) investigated changes in the connectivity of prefrontal and more posterior brain areas between 2nd and 3rd grade using a cross-sectional approach. They observed differential functional connectivity between left DLPFC and posterior brain areas. In particular, changes in functional connectivity between 2nd and 3rd grade were stronger in what the authors termed dorsal (superior parietal lobe, AG) as compared to ventral stream areas (parahippocampal gyrus, lateral occipital cortex, lingual gyrus).

Krueger et al. (2011) used multivariate Granger causality to evaluate *effective* connectivity in adult numerical cognition. Multivariate Granger causality mapping not only quantifies the co-activation of two brain regions for a given task, but also allows one to assess the direction of the connections between the respective areas. The authors observed a fronto-parietal network for multiplication, involving a reciprocal parietal IPS-IPS circuit which subserves number magnitude information. This magnitude processing related network was also interlaced with a reciprocal fronto-parietal circuit from the dorsolateral prefrontal cortex and the IPS associated with the execution and updating of arithmetic operations. Importantly, the parietal cortex received more inputs from the frontal cortex than the other way around, indicating the central role of the parietal cortex in number processing.

**Figure 1:**
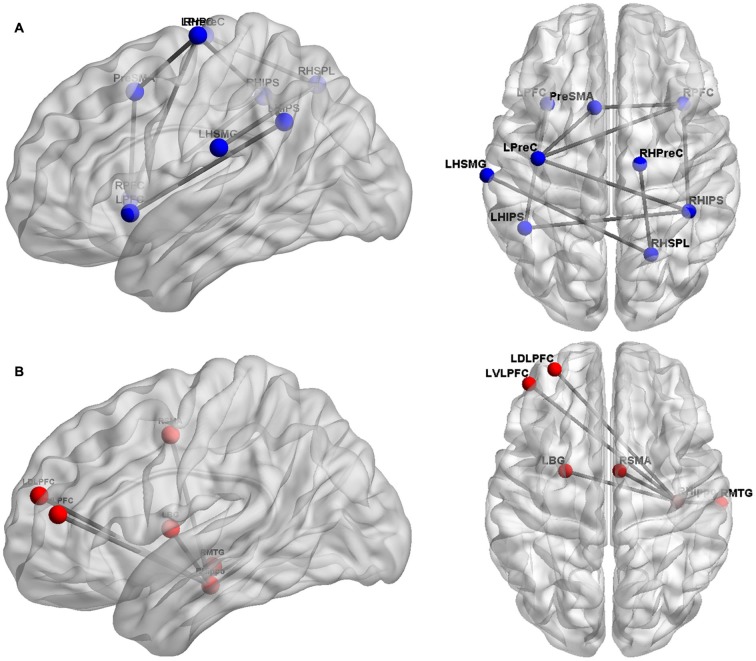
**Overview of cortical sites considered in the studies evaluating functional/effective brain connectivity**. Panel **(A)** depicts the cortical sites (blue spheres) and their functional/effective connectivity patterns (gray lines), found by Krueger et al. (2011), Emerson and Cantlon (2012), and Park et al. (2013, 2014). These studies primarily focused on the fronto-parietal network of numerical cognition. Panel **(B)** shows the cortical regions (red spheres) and their functional/effective connectivity patterns (gray lines) identified by Supekar et al. (2013) found to be predictive of numerical learning.

Another method to evaluate effective connectivity is the approach of psychophysical interaction analysis (PPI), as used by Park et al. (2013, 2014, see also Cho et al., 2012; Qin et al., 2014). For adults, Park et al. (2013) used custom-made reaction time experimental tasks assessing (i) non-symbolic addition and subtraction, (ii) number matching as well as (iii) shape matching. They found increased effective connectivity within the right parietal cortex as well as between the right and left parietal cortices for arithmetic tasks in general and subtraction in particular. Importantly, the degree of effective connectivity was associated positively with behavioral performance in the subtraction task. Furthermore, Park et al. (2014) investigated effective connectivity of the right parietal cortex with the left supramarginal gyrus and the right precentral gyrus in 4–6-year-old children. The degree of connectivity from the right parietal cortex to the right precentral gyrus was predictive of performance on a standardized symbolic math test (see Figure 1 for an overview of the connections suggested by functional/effective connectivity analyses). Using the same method, Rosenberg-Lee et al. (2015) investigated differences in functional connectivity of the IPS between typically developing 7–9-year-old children and a sample of children from the same age group with DD. Interestingly, the authors found what they called hyperconnectivity of the bilateral IPS in children with DD with ventro- and dorsolateral PFC as well as the SMG. The authors attributed this phenomenon to involvement of compensatory mechanisms. On the other hand, they also suggested that the “engagement of these circuits may result in the activation of problem-irrelevant information that in turn disrupts problems solving” (p. 18, see also below for findings on structural connectivity in children with DD).

The conclusion of Supekar et al. (2013), stating that hippocampal-prefrontal connectivity is specifically associated with numerical learning, was corroborated by an evaluation of effective connectivity of the hippocampus. Using PPI, Cho et al. (2012) revealed strong causal bidirectional connectivity between the right hippocampus and the left VLPFC and DLPFC to be associated with the application of retrieval-based solution strategies to an addition task in 7–10-year-old children. The influence of hippocampal-prefrontal connectivity on numerical learning was further specified in a paper by Qin et al. (2014), which so far represents the only longitudinal study investigating the influence of brain connectivity on numerical development. The authors observed that the connectivity of the hippocampus with prefrontal and parietal cortices was predictive of the gain in 7–9-year-old children’s fact retrieval fluency in mental addition over a period of more than 1 year. Thus, numerical development seems to be associated with changes in hippocampal-neocortical connectivity.

Summarizing the results of studies evaluating functional and effective connectivity associated with numerical cognition clearly indicates that number processing involves a widespread network including (intra)parietal (e.g., IPS, SPL, SMG, AG) but also (pre)frontal cortex sites (e.g., DLPFC, VMPFC, preCG, SMA, IFG) as well as the hippocampus. The latter seems to be particularly involved in numerical learning and development because hippocampal-prefrontal as well as hippocampal-parietal connectivity is reliably associated with children’s use of more sophisticated retrieval-based solution strategies in mental arithmetic. Additionally, the strength of fronto-parietal connectivity was associated with better math proficiency. In line with the TCM these analyses of functional and effective connectivity provide converging evidence for numerical cognition to be subserved by a fronto-parietal network also incorporating hippocampal structures. However, those analyses do not allow for the identification of anatomical WM fiber tracts connecting brain areas with correlated brain activations. This can only be achieved by considering structural connectivity. In the following, we describe another set of studies which evaluated structural connectivity in two different ways to pinpoint the WM tracts involved in numerical cognition.

### Structural Connectivity

There are two different approaches to investigate structural connectivity (see Table 2 for an overview of studies evaluating structural connectivity). The first is to evaluate the correlation of diffusion parameters in predefined ROI with either behavioral performance or with fMRI activation peaks observed for numerical tasks. Fractional anisotropy (FA) and/or radial diffusion (RD; see Mukherjee et al., 2008 for an explanation of the physical principles) are often used diffusion parameters in these analyses. Additionally, ROIs are usually located to reflect a specific (mostly) atlas-identified WM pathway. The second approach is fiber tractography, which allows for the virtual reconstruction of entire WM pathways. Thereby, diffusion tensor tractography can characterize not only the orientation but also the integrity of WM fibers *in vivo* and noninvasively (Basser et al., 1994). The following section will discuss studies using atlas-based ROI analyses and fiber tractography in turn.

#### Atlas-based ROI Analyses of Diffusion Measures

Studies using ROI analyses provided evidence for the involvement of anterior to posterior association and projection fiber tracts in numerical cognition. With respect to *anterior to posterior association fiber tracts*, Rykhlevskaia et al. (2009) observed that increased FA in a temporo-parietal ROI incorporating parts of the superior longitudinal fasciculus (SLF), the inferior longitudinal fasciculus (ILF), and the inferior fronto-occipital fasciculus (IFOF) was associated with better performance of 7–9-year-old children in the arithmetic subtest of an IQ test. The importance of fronto-parietal connections was further corroborated by Tsang et al. (2009). These authors used a combination of tests administered outside and inside the scanner, to investigate the association between FA in a central part of the SLF and performance in a computerized approximate arithmetic task. In particular, the authors considered performance scores of 10–15-year-olds from the arithmetic subtest of a scholastic achievement test to control for the specificity of their results. They found an association of higher FA in the SLF and better performance in approximate arithmetic, indicating the importance of fronto-parietal connectivity for performance in mental arithmetic.

*As regards projection fiber tracts*, Rykhlevskaia et al. (2009) reported that increased FA in a temporo-parietal ROI incorporating parts of the anterior thalamic radiation and the cortico-spinal tract was associated with better performance of 7–9-year-old children in the arithmetic subtest of an IQ test. Comparably, van Eimeren et al. (2008) used ROI analyses to investigate the association of WM connectivity with arithmetic performance. They found that in 7–9-year-old children increasing FA in ROIs from the superior corona radiata (SCR) was associated with better performance in the arithmetic subtests of an IQ test administered outside the scanner. To a lesser degree this also held true for FA in ROIs from the ILF. van Eimeren et al. (2010) also found evidence for an involvement of the SCR in numerical cognition. They observed that higher FA in a ROI reflecting a central segment of the left SCR was associated reliably with a stronger BOLD response in the left AG, as recorded during retrieval-prone calculations in a sample of mostly university students.

Other studies reported a* combination of projection and association fiber tracts* to be recruited in numerical cognition. Matejko et al. (2013), using tract-based spatial statistics (TBSS), found higher FA in the left SLF, SCR, and cortico-spinal tract of 17 to 18-year-olds to be associated with better performance in the arithmetic subtest of a scholastic achievement test. TBSS (see also Hu et al., 2011; Li et al., 2013b) employs a voxel-wise statistic followed by the projection onto an alignment-invariant mean FA skeleton in order to derive clusters, in which FA correlates with a dependent variable. These clusters may be but do not necessarily need to be used for ROI analyses. Instead, most other studies reported in this paragraph extracted FA for ROIs in tracts of interest (after atlas-based identification) from aligned and spatially smoothed diffusion imaging data (Jones et al., 2005).

There is also another approach to investigate structural connectivity in numerical cognition. Instead of associating diffusion measures in specified ROIs with performance or GM activation, another subset of studies evaluated differences in ROI-based diffusion parameters between different populations. Rykhlevskaia et al. (2009) found reduced FA in ROIs located in the IFOF, ILF, SLF, amongst others, in children with developmental DD, as compared to typically developing children. Recent data by Kucian et al. (2014) substantiated impairments in WM connectivity as indicated by reduced FA and RD in a posterior part of the SLF, in particular in children with DD. These authors suggested that DD may be considered a disconnection syndrome (see also Klein et al., 2013a for the case of acquired acalculia). Navas-Sánchez et al. (2014), when studying math-gifted adolescents, observed higher WM integrity, as indicated by higher FA in ROIs located in the SLF adjacent to inferior parietal cortex areas. Barnea-Goraly et al. (2005a) found that reduced arithmetic competencies in velocardiofacial syndrome may be caused by structural WM aberrations in inferior parietal cortex (see also Lebel et al., 2010; Till et al., 2011, for WM differences associated with impaired numerical performance in children with fetal alcohol spectrum disorder and youths with multiple sclerosis, respectively).

Finally, there were another two studies specifically investigating the influence of the duration of abacus use on brain connectivity. Li et al. (2013b) employed TBSS and found increased FA in the left callosal forceps major only, whereas Hu et al. (2011) observed increased RD for abacus users after three years of abacus training in a variety of WM connections, including “the internal capsule (IC), corona radiata and posterior thalamic radiation” (p. 19) as well as the SLF.

In summary, ROI analyses of DTI data are commonly used to detect diffusion parameter alterations, as measured by FA values. However, it is worth noting that WM tracts specified by these analyses are simply those that pass through the respective ROI, as indicated by comparison with a brain atlas. Importantly, this means that the specified tracts were not identified directly to connect to cortex sites of interest. Fiber tractography based on task-related fMRI data, however, enables the virtual reconstruction of WM pathways connecting cortex areas found or assumed to be included in s processing model such as the TCM.

#### Fiber Tractography

A last set of studies interested in structural connectivity used either probabilistic or deterministic fiber tractography to identify WM connections. Fiber tractography aims at delineating WM pathways involved by virtually reconstructing the most probable WM tract pathways between user-defined seed points. Probabilistic tracking differs from deterministic tracking in that the probability for false negative reconstructions of specific tracts is taken into account. This probability is typically elevated in areas where fibers cross, merge or kiss. By employing both probabilistic and deterministic fiber tracking, Rykhlevskaia et al. (2009) compared the brain connectivity pattern in children with and without developmental DD. The authors found that typically developing children showed more inter-hemispheric (superior parietal) connectivity as well as stronger connectivity of the right temporal-parietal cortex. Probabilistic and deterministic fiber tracking analyses linking WM and GM alterations in children with developmental DD “point to tracts connecting the fusiform gyrus with temporal-parietal WM, most likely via the ILF, as a major locus of neuroanatomical abnormalities in DD” (Rykhlevskaia et al., 2009, p. 11).

Cantlon et al. (2011) employed deterministic fiber tracking to further investigate the influence of inter-hemispheric IPS to IPS connectivity on number processing in typically developing children. The authors observed that FA within tracked fibers of the left isthmus of the corpus callosum was correlated positively with performance in a number magnitude comparison task administered in the scanner in six-year-old children. On the other hand, Li et al. (2013a) used probabilistic fiber tracking to identify and differentiate the course of the different anterior to posterior association fiber tracts. Using TBSS, FA values of the WM tracts identified were correlated with children’s performance in the arithmetic subtest(s) of an IQ test. Reliable positive associations between FA values and arithmetic performance were observed for the left SLF, ILF and bilateral IFOF.

More recent studies using probabilistic or deterministic fiber tracking have tried to integrate the identified pathways for numerical cognition into the broader architecture of dorsal and ventral processing streams, as previously done for other cognitive domains such as language (e.g., Hickok and Poeppel, 2007; Rauschecker and Scott, 2009; Weiller et al., 2011). Klein et al. (2013b) investigated WM connections between seed points observed to be activated in either more difficult (calculation-based) or more easy (retrieval-based) addition problems. For both conditions the authors reconstructed pathways encompassing the SLF and the external/extreme capsule (EC/EmC) system indicating that both magnitude- and fact retrieval-related processing were subserved by two largely distinct networks, both of them comprising dorsal and ventral connections. This distinction between magnitude- and fact retrieval-related processing on the level of structural brain connectivity was further substantiated by the results of Klein et al. (2014). These authors showed that the proposed differentiation generalizes to other numerical tasks (i.e., number bisection and exact/approximate addition). This indicates that magnitude- and fact retrieval-related processing may indeed rely on different neural networks, even though these networks operate in an integrated manner to solve numerical tasks most efficiently. This is also in line with the results of Van Beek et al. (2014). These authors found that higher FA in the left anterior portion of the arcuate fasciculus specifically predicted better addition and multiplication but not subtraction and division performance. As the arcuate fasciculus links frontal with temporo-parietal cortex sites the authors argue that “the association between the left arcuate fasciculus-anterior and addition/multiplication reflects involvement of phonological processing” (p. 117) related to arithmetic fact retrieval.

Finally, Willmes et al. (2014) observed a common ventral fronto-parietal connection encompassing the EC/EmC system for the general cognitive operation of *semantic classification* for the domains of language (e.g., word/non-word decisions) and number processing (e.g., odd/even judgments). Interestingly, this network appeared to be augmented by a dorsal connection to the IPS running along the SLF, when number magnitude was decision relevant, as is the case in number magnitude comparison.

In summary, analyses of structural connectivity underlying numerical cognition indicate crucial involvement of both association fiber tracts running from anterior to posterior (e.g., SFL, EC/EmC system, ILF, and IFOF) as well as projection fiber tracts (e.g., SCR, thalamic radiation) and transcallosal commissural fibers connecting the bilateral intraparietal sulci. Comparable to the results of studies investigating functional/effective connectivity, the findings from structural connectivity analyses corroborate the proposition of a fronto-parietal network subserving numerical cognition. In particular, the SLF and the EC/EmC system constitute important fronto-parietal pathways connecting number-specific areas in the parietal cortices (e.g., IPS, AG) with number unspecific areas in (pre)frontal cortex (e.g., DLPFC, IFG), as proposed by the TCM.

### White Matter Pathways in Numerical Cognition

**Figure 2:**
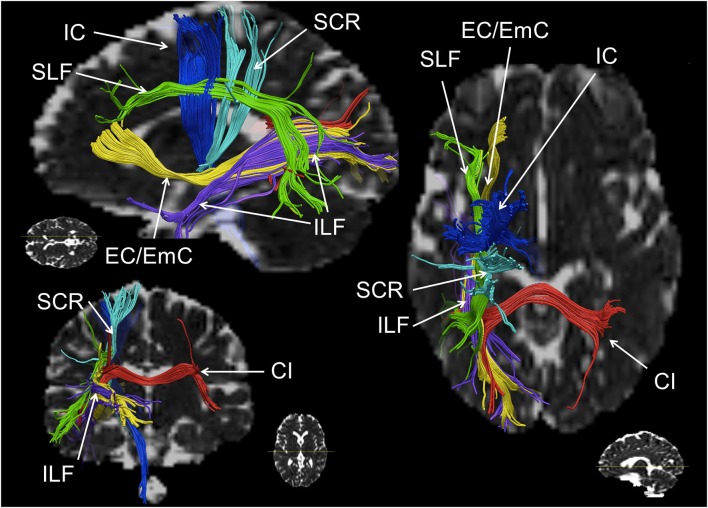
**Schematic reconstruction of association (green, yellow), projection (blue) and commissural (red) fiber tracts repeatedly observed in numerical cognition tasks (in axial, sagittal and coronal orientation)**. The superior longitudinal fasciculus (SLF) is displayed in green, the inferior longitudinal fasciculus (ILF) and the external/extreme capsule (EC/EmC) system are depicted in yellow, parts of the internal capsule (IC) in dark blue, the superior coronar radiata (SCR) is shown in light blue, and interhemispheric parietal to parietal connections encompassing the callosal isthmus (CI) are shown in red. Virtual dissections were performed for one individual with seed regions chosen deliberately for illustration purposes only, regarding white matter (WM) pathways involved in numerical cognition.

There is considerable convergence with respect to the fronto-parietal WM pathways connecting domain-specific number-related parietal brain areas (IPS, AG) to more domain-general (pre)frontal areas. The SLF and the EC/EmC system were identified repeatedly to be associated with fronto-parietal processing in numerical cognition (e.g., Rykhlevskaia et al., 2009; Tsang et al., 2009; van Eimeren et al., 2010; Klein et al., 2013a,b; Matejko et al., 2013; Kucian et al., 2014; Navas-Sánchez et al., 2014; see Figure 2 for a schematic illustration). Importantly, the association of fronto-parietal connectivity with numerical performance encompassing these systems is not only in line with the results of the functional connectivity analyses described above, but also corroborates the propositions of the TCM. Furthermore, involvement of projection fibers such as the (superior) corona radiata, possibly connecting motor cortices and subcortical structures such as the thalamus, were frequently observed to be involved in numerical cognition (van Eimeren et al., 2008, 2010; Rykhlevskaia et al., 2009; Hu et al., 2011).

The importance of the SCR is hard to reconcile with the results of functional connectivity analyses, which usually did not consider cortico-subcortical connections or subcortical structures. Nevertheless, the involvement of these projection fibers is in line with propositions of earlier versions of the TCM by Dehaene and Cohen (1995, 1997), which have not been pursued systematically so far—possibly due to the cortex-centerd focus of recent (fMRI) research on numerical cognition.

**Figure 3:**
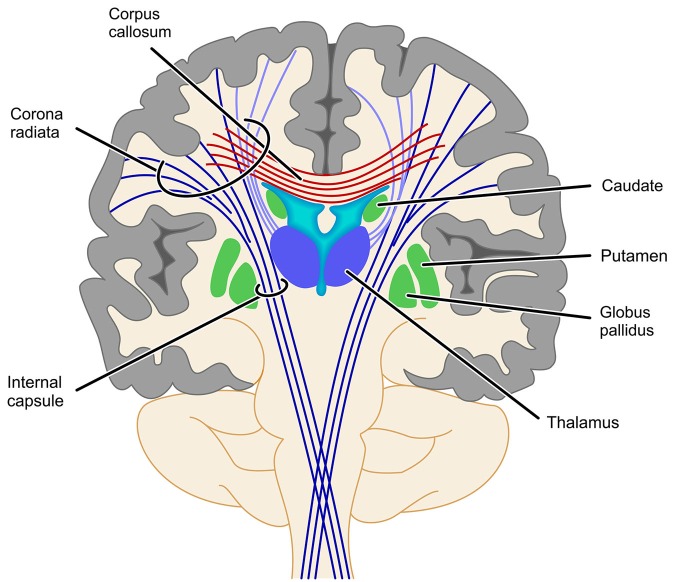
**Schematic illustration of problems with the identification of projection fibers**. Fiber tracts identified by atlas-based ROI analyses depend strongly on where exactly along this bundle of tracts the respective ROI is placed. As most of the ROIs reviewed in this article placed their ROI somewhere between the basal ganglia and the cortex it is obvious that such a ROI might well involve fibers of the SCR, the cortico-spinal tract/IC, the thalamic radiation (light purple) or even commissural fibers (cortico-striatal tract not depicted for reasons of clarity).

With respect to the involvement of the SCR it is interesting that fiber tracts, which have been found to be involved in number processing less consistently, seem to be closely related neuro-anatomically. In particular, the cortico-spinal tract (Matejko et al., 2013; investigated in some cases at the level of the internal capsule (Hu et al., 2011; Navas-Sánchez et al., 2014) and the right thalamic radiation (Rykhlevskaia et al., 2009; Hu et al., 2011) are often aggregated to constitute the SCR. Neuro-anatomically the cortico-spinal tract, but also the superior peduncle of the thalamic radiation are part of the corona radiata—as is the SCR. Therefore, functional involvement of the SCR seems particularly reasonable from a theoretical point of view. The thalamic radiation directly connects the cortex with the ventrolateral thalamus, while the cortico-striatal tract connects the cortex indirectly with the ventrolateral thalamus via the striatum. Thus, these structures connect GM areas known to be involved in numerical cognition either directly or indirectly (via the basal ganglia) with the thalamus. Interestingly, early versions of the TCM (Dehaene and Cohen, 1995, 1997) suggested a vital role of the basal ganglia and the thalamus in numerical cognition. On the other hand, assuming the involvement of the cortico-spinal tract or the IC to be associated with processing of numerical information is less evident, because for both structures connections to the parietal lobes are strongly associated with motor and somatosensory processes (e.g., Newton et al., 2006; Lotze et al., 2011; see Catani et al., 2012 for an overview). However, all evidence for an involvement of these different parts of the corona radiata comes from studies specifying the involved WM in an atlas-based approach (either ROI analyses, e.g., Rykhlevskaia et al., 2009; Navas-Sánchez et al., 2014 or TBSS, e.g., Hu et al., 2011; Matejko et al., 2013). We suggest that any strong interpretation of the functional involvement of the SCR, the thalamic radiation, the cortico-spinal tract, the cortico-striatal tract and even commissural fibers should be made with great care, because the atlas-based identification of WM tracts highly depends on where exactly the respective ROI is placed (see Figure 3 for a schematic illustration). Generally, ROI analyses do not provide virtual reconstructions of the WM tracts connecting two GM areas associated with number processing. Therefore, the WM tracts identified by ROI analyses reflect all tracts passing through the respective ROI, instead of considering only those tracts connecting the WM areas of interest as in fiber tractography. For the present WM tracts (i.e., SCR, cortico-spinal tract/IC, thalamic radiation, cortico-striatal tract, and commissural fibers) a respective ROI may incorporate fibers of more than one tract, and small variation in the location of the ROI may easily change the involved fiber tracts. It is even easily possible to capture *all* of the latter tracts in one atlas-based ROI. Importantly, this variability of results can be reduced by employing methods of fiber tractography, which makes them highly desirable for more studies evaluating brain connectivity in numerical cognition. While studies on functional and atlas-based structural connectivity paved the ground for a more general understanding of the interaction of WM with GM in numerical cognition, the actual connectivity within the fronto-parietal network of numerical cognition seems to be captured better using tractography-based analyses.

Generally, there seems to be notable agreement regarding the identified fronto-parietal WM tracts, but so far there is no coherent picture with respect to lateralization of the tracts involved. Some studies reported bilateral hemispheric connections associated with number processing (e.g., Rykhlevskaia et al., 2009; Hu et al., 2011; Klein et al., 2013b), whereas others only found significant results for left hemispheric fiber tracts (e.g., van Eimeren et al., 2008; Tsang et al., 2009; Kucian et al., 2014; Matejko et al., 2013) or deliberately chose to focus on the left hemisphere, because the authors were specifically interested in verbal numerical representations supposed to be left-lateralized (e.g., Klein et al., 2013a, b; Willmes et al., 2014). As regards interhemispheric connections, only Cantlon et al. (2011) directly compared inter-hemispheric (intra)parietal to (intra)parietal connections and found them to be reliable for the genu, isthmus and posterior splenium tracts of the corpus callosum. Additionally, WM tracts along the forceps major (e.g., Rykhlevskaia et al., 2009; Klein et al., 2013a; Li et al., 2013a,b) were observed to be involved in numerical cognition connecting (intra)parietal areas.

Finally, other WM pathways have been found to be involved in number processing less consistently. In particular, involvement of the IFOF (e.g., Rykhlevskaia et al., 2009; Li et al., 2013a), connecting frontal cortex sites with occipital sensory cortical areas was reported. Since all reported studies used visually presented stimuli this most probably reflects involvement of visual perceptual processes in numerical cognition. Additionally, the ILF was repeatedly found to be associated with numerical performance (e.g., van Eimeren et al., 2008; Rykhlevskaia et al., 2009; Li et al., 2013a). As the ILF represents a parieto-temporal connection, it may be most likely involved in connecting left-hemispheric perisylvian language areas, as proposed to be associated with the verbal representation of numbers in the TCM.

### Other Methodological Limitations and Implications

Substantial convergence concerning the involvement of WM pathways in numerical cognition is notable, considering the variety of methodological approaches and tasks employed to investigate quite different populations. The different methods for investigating brain connectivity come with specific advantages and limitations. Functional connectivity analyses (e.g., Emerson and Cantlon, 2012) can only provide temporal correlations between activation in remote brain areas. Effective connectivity analyses (e.g., Krueger et al., 2011; Cho et al., 2012) additionally specify the direction in which one neuronal system exerts an influence over another. However, both attempts do not allow identification of WM tracts in a strict sense. As regards structural connectivity, ROI analyses of DTI data (e.g., van Eimeren et al., 2008, 2010; Matejko et al., 2013) require *a priori* hypotheses about the WM tracts involved. Additionally, intra- and inter-individual variability in the delineation of ROIs limits their reproducibility and reliability, as discussed above regarding involvement of the SCR. On the other hand, the interpretation of results from probabilistic and deterministic tractography (e.g., Rykhlevskaia et al., 2009; Willmes et al., 2014) depends on placement and size of the seed regions as well as on algorithm settings (Aoki et al., 2007). Additionally, tractography is particularly complex for regions where fibers cross, kiss, or merge, possibly leading to artefactual reconstructions (Basser et al., 2000). Furthermore, numerical performance was not only assessed inside (e.g., Tsang et al., 2009; Cantlon et al., 2011) but also outside (e.g., Rykhlevskaia et al., 2009; Matejko et al., 2013) the scanner. Moreover, assessment procedures also ranged from standardized diagnostic test instruments (e.g., TEMA, WIAT) over specific subtests selected from those standardized tests (e.g., subtest numerical operations from the WIAT; e.g., Barnea-Goraly et al., 2005a; Rosenberg-Lee et al., 2011; Emerson and Cantlon, 2012) to custom-made experimental reaction time tasks (e.g., Krueger et al., 2011; Park et al., 2013).

Finally, about half of the studies evaluated WM connectivity in children up to the age of 11 years (e.g., Emerson and Cantlon, 2012; Park et al., 2014), another three included adolescents between 11 and 18 years of age (e.g., Matejko et al., 2013; Navas-Sánchez et al., 2014), and only five studies investigated healthy adults (mostly students, e.g., Krueger et al., 2011; Park et al., 2013). Furthermore, there was one single-case stroke patient study (Klein et al., 2013a).

This shows that a considerable number of studies on brain connectivity in numerical cognition has been conducted with children and adolescents. Therefore, it seems important to evaluate whether these data shed new light on the potential development of WM connectivity within the fronto-parietal network underlying numerical cognition. However, no specific systematic trend was evident for age. Fronto-parietal associations (e.g., SLF) as well as projecting tracts (e.g., SCR) were found to be involved in numerical cognition from the youngest ages studied. This finding may be expected, because the identified fiber tracts (e.g., ILF, SLF, SCR, etc.) are important neuro-anatomical structures, which develop independently from specific cognitive functions during childhood and adolescence (e.g., Barnea-Goraly et al., 2005b; Huang et al., 2006; Asato et al., 2010; see Peters et al., 2012 for a recent meta-analysis).

Against this background, future studies are needed to evaluate how far the functional coupling of particular cortex areas or WM pathways associated with numerical cognition are specific to number processing and/or calculation. The study of Willmes et al. (2014) provides a first step in this direction. The authors investigated the WM connections associated with the general cognitive operation of *semantic classification* across the domains of language and number processing. They observed a common fronto-parietal connection encompassing the EC/EmC system for semantic classification, irrespective of content. This is in line with evidence suggesting that functional specificity of GM cortex areas (e.g., as suggested by Brodmann, 1909) is hard to reconcile with the differential involvement of cortical areas in a variety of tasks. For instance, Simon et al. (2002, 2004; see also Humphreys and Lambon Ralph, 2014 for a recent fMRI meta-analysis over 8 cognitive domains including number processing) found that the IPS was activated not only in number processing but also (partly overlapping) for the initiation of saccades, attention shifting, grasping, pointing, and language processing. This corroborates the notion of systemic localization, as suggested early on by Reinvang (1985). In this view, domain specificity may not be a question of localization (which cortex areas?) or connectivity (which fiber tracts?). Instead, it is important to concomitantly consider which cortex areas are connected to which other areas by which fiber tracts. The particular combination of cortex areas connected by specific fiber tracts may then be an indicator of domain-specificity.

Moreover, the question of how numerical learning and development manifest in WM connectivity is of particular interest. There is evidence for qualitative changes in GM activation patterns following numerical learning (e.g., Delazer et al., 2003; Kaufmann et al., 2011). There are now indications that learning may change WM connectivity parameters (Sagi et al., 2012; see Menon, 2013 for implications on cognitive development). For numerical cognition, Supekar et al. (2013) were able to show that numerical learning is specifically predicted by the connectivity of the hippocampus with prefrontal cortex sites (see Figure 1, see also Cho et al., 2012). Additionally, Qin et al. (2014) found longitudinal gains for fact retrieval fluency in 7–9-year-old children in mental addition, which were predicted by effective connectivity of the hippocampus with prefrontal and parietal cortices. Thus, numerical development as characterized by increasing use of retrieval-based solution strategies seems to be associated specifically with changes in hippocampal-neocortical connectivity. In the future, it might also be interesting to study the development of the different networks underlying arithmetic fact retrieval and number magnitude processing as identified by Klein et al. (2013a).

In addition to variation by age, there are five studies investigating brain connectivity in special populations: children with DD (Rykhlevskaia et al., 2009; Kucian et al., 2014; Rosenberg-Lee et al., 2015), individuals with velocardiofacial syndrome (Barnea-Goraly et al., 2005a), and math-gifted children (Navas-Sánchez et al., 2014). Molko et al. (2004) also provide first evidence regarding structural alterations in fiber orientation in children with Turner syndrome, who often present with numerical deficits.

## Synthesis and Perspectives

Considering the variety of methodological approaches, types of assessment instruments, and populations investigated, the convergence of evidence regarding brain connectivity in numerical cognition is remarkable. Analyses of both functional/effective as well as structural connectivity consistently corroborate the propositions of the TCM: Numerical cognition seems to be subserved by a widespread network including (intra)parietal (e.g., IPS, SPL, SMG, AG) but also (pre)frontal cortex sites (e.g., DLPFC, VMPFC, preCG, SMA, IFG), as well as the hippocampus. Studies on functional/effective connectivity indicate a specific role of the hippocampus in numerical development. In children, hippocampal-prefrontal as well as hippocampal-parietal connectivity were found to be associated with the acquisition of retrieval-based solution strategies, while in adults hippocampal-parietal connectivity was associated with the retrieval of arithmetic facts. On the other hand, analyses of structural connectivity provide converging evidence for functional involvement of association fibers from the SLF dorsally as well as the EC/EmC system ventrally as the primary fronto-parietal connections in the numerical cognition network. Synced with commissural fibers such as inter-hemispheric IPS to IPS connections running transcallosally along the callosal isthmus and the forceps major, all these results further corroborate the proposition of the TCM of numerical cognition being subserved by fronto-parietal neural networks.

The specifications in this review of the WM pathways involved in numerical cognition also extend the TCM with respect to how GM areas associated with specific number-related representations (e.g., IPS: number magnitude vs. AG: arithmetic facts) may work together. The involvement of projection fibers such as the SCR (in particular the part of the thalamic radiation and the cortico-striatal tract) may revive the importance of the basal ganglia as well as the thalamus, which were already incorporated in early versions of the TCM by Dehaene and Cohen (1995, 1997). As a consequence, it is now possible to evaluate the idea that numerical impairments arise from WM disconnections between specific cortical areas in an individual brain (Kucian et al., 2014 for developmental DD; Klein et al., 2013a for acquired acalculia). This is in line with considerations for deficits in other domains (e.g., Schlaug et al., 2009 for aphasia and Rusconi et al., 2009 for a disconnection account of the Gerstmann syndrome).

These new insights into the WM correlates of numerical cognition also come with methodological as well as theoretical implications for future studies on brain hodology underlying numerical cognition. On the methodological side it would be interesting to combine different approaches to obtain a more comprehensive picture of the neural WM and GM correlates of numerical cognition. For instance, functional and structural connectivity analyses may be complemented by fMRI and voxel-based morphometry (see Rykhlevskaia et al., 2009, for a first attempt). Additionally, the ultrahigh-resolution 3D-model of the human brain (“BigBrain”) provides unprecedented information on the interconnection of cortical regions (Amunts et al., 2013). Taken together, considering brain connectivity not only seems inevitably mandatory in order to understand human numerical cognition but it also opens up new avenues for future research.

## Conflict of Interest Statement

The Review Editor Wolfgang Grodd declares that, despite being affiliated to the same institution as authors Klaus Willmes and Elise Klein, the review process was handled objectively and no conflict of interest exists. The authors declare that the research was conducted in the absence of any commercial or financial relationships that could be construed as a potential conflict of interest.

## References

[B1] Amunts K. Lepage C. Borgeat L. Mohlberg H. Dickscheid T. Rousseau M.-E.. (2013). BigBrain: an ultrahigh-resolution 3D human brain model. Science 340, 1472–1475. 10.1126/science.1235381 23788795

[B2] Aoki S. Masutani Y. Abe O. (2007). Magnetic resonance diffusion tractography in the brain—Its application and limitation. Brain Nerve 59, 467–476. 17533972

[B3] Asato M. R. Terwillinger R. Woo J. Luna B. (2010). White matter development in adolescence: a DTI study. Cereb. Cortex 20, 2122–2131. 10.1093/cercor/bhp282 20051363 PMC2923214

[B4] Barnea-Goraly N. Eliez S. Menon V. Bammer R. Reiss A. L. (2005a). Arithmetic ability and parietal alterations: a diffusion tensor imaging study in velocardiofacial syndrome. Brain Res. Cogn. Brain Res. 25, 735–740. 10.1016/j.cogbrainres.2005.09.013 16260124

[B5] Barnea-Goraly N. Menon V. Eckert M. Tamm L. Bammer R. Karchemskiy A.. (2005b). White matter development during childhood and adolescence: a cross-sectional diffusion tensor imaging study. Cereb. Cortex 15, 1848–1854. 10.1093/cercor/bhi062 15758200

[B78] Bartolomeo P. De Schotten M. T. Chica A. B. (2012). Brain networks of visuospatial attention and their disruption in visual neglect. Front. Hum. Neurosci. 6:110. 10.3389/fnhum.2012.00110 22586384 PMC3343690

[B7] Basser P. J. Mattiello J. Le Bihan D. (1994). MR diffusion tensor spectroscopy and imaging. Biophys J. 66, 259–267. 10.1016/S0006-3495(94)80775-1 8130344 PMC1275686

[B6] Basser P. J. Pajevic S. Pierpaoli C. Duda J. Aldroubi A. (2000). In vivo fiber tractography using DT-MRI data. Magn. Reson. Med. 44, 625–632. 10.1002/1522-2594(200010)44:4<625::aid-mrm17>3.0.co;2-o 11025519

[B8] Broca P. (1861). Remarques sur le siege de la faculté du langage articulé, suives d’une observation d’aphemie. Bull. Mem. Soc. Anatomique Paris 2, 330–357.

[B9] Brodmann K. (1909). Vergleichende Lokalisationslehre der Großhirnrinde. Leipzig: J. A: Barth.

[B79] Campbell F. (1905). Histological Studies on Localization of Cerebral Function. Cambridge: Cambridge University Press.

[B10] Cantlon J. F. Davis S. W. Libertus M. E. Kahane J. Brannon E. M. Pelphrey K. A. (2011). Intra-parietal white matter development predicts numerical performance in children. Learn Individ Differ. 21, 672–680. 10.1016/j.lindif.2011.09.003 22180720 PMC3240671

[B12] Catani M. Dell’Acqua F. Bizzi A. Forkel S. J. Williams S. C. Simmons A.. (2012). Beyond cortical localization in clinico-anatomical correlation. Cortex 48, 1262–1287. 10.1016/j.cortex.2012.07.001 22995574

[B80] Catani M. De Schotten M. T. Slater D. Dell’Acqua F. (2013). Connectomic approaches before the connectome. Neuroimage 80, 2–13. 10.1016/j.neuroimage.2013.05.109 23735262

[B11] Catani M. Ffytche D. (2005). The rise and fall of disconnection syndromes. Brain 128, 2224–2239 10.1093/brain/awh622 16141282

[B13] Cho S. Metcalfe A. W. S. Young C. B. Ryali S. Geary D. C. Menon V. (2012). Hippocampal-prefrontal engagement and dynamic causal interactions in the maturation of children’s fact retrieval. J. Cogn. Neurosci. 24, 1849–1866. 10.1162/jocn_a_00246 22621262 PMC3462165

[B14] Cohen L. Dehaene S. (1995). Number processing in pure alexia: the effect of hemispheric asymmetries and task demands. Neurocase 1, 121–137 10.1093/neucas/1.2.121-a

[B81] College Board USA (2006). Preliminary Scholastic Aptitude Test.

[B15] Conturo T. E. Lori N. F. Cull T. S. Akbudak E. Snyder A. Z. Shimony J. S.. (1999). Tracking neuronal fiber pathways in the living human brain. Proc. Natl. Acad. Sci. U S A 96, 10422–10427. 10.1073/pnas.96.18.10422 10468624 PMC17904

[B16] Dehaene S. Cohen L. (1995). Towards an anatomical and functional model of number processing. Math. Cogn. 1, 83–120.

[B83] Dehaene S. Cohen L. (1997). Cerebral pathways for calculation: double dissociation between rote verbal and quantitative knowledge of arithmetic. Cortex 33, 219–250. 10.1016/s0010-9452(08)70002-9 9220256

[B17] Dehaene S. Piazza M. Pinel P. Cohen L. (2003). Three parietal circuits for number processing. Cogn. Neuropsychol. 20, 487–506. 10.1080/02643290244000239 20957581

[B18] Delazer M. Domahs F. Bartha L. Brenneis C. Lochy A. Trieb T.. (2003). Learning complex arithmetic–a fMRI study. Brain Res. Cogn. Brain Res. 18, 76–88. 10.1016/j.cogbrainres.2003.09.005 14659499

[B19] Emerson R. W. Cantlon J. F. (2012). Early math achievement and functional connectivity in the fronto-parietal network. Dev. Cogn. Neurosci. 25, S138–S151. 10.1016/j.dcn.2011.11.003 22682903 PMC3375498

[B84] Forkel S. J. De Schotten M. T. Dell’Acqua F. Kalra L. Murphy D. G. Williams S. C.. (2014). Anatomical predictors of aphasia recovery: a tractography study of bilateral perisylvian language networks. Brain 137, 2027–2039. 10.1093/brain/awu113 24951631

[B20] Ginsburg H. P. Baroody A. J. (2003). Test of Early Mathematics Ability. 3rd Edn. Austin, TX: Pro-Ed.

[B21] Henschen S. E. (1920). Klinische und Anatomische Beitrage zur Pathologie des Gehirns. Pt 5. Stockholm: Nordiska Bukhandeln.

[B22] Hickok G. Poeppel D. (2007). The cortical organization of speech processing. Nat. Rev. Neurosci. 8, 393–402. 10.1038/nrn2113 17431404

[B23] Hu Y. Geng F. Tao L. Hu N. Du F. Fu K.. (2011). Enhanced white matter tracts integrity in children with abacus training. Hum. Brain Mapp. 32, 10–21. 10.1002/hbm.20996 20235096 PMC6870462

[B24] Huang H. Zhang J. Wakana S. Zhang W. Ren T. Richards L. J.. (2006). White and gray matter development in human fetal, newborn and pediatric brains. Neuroimage 33, 27–38. 10.1016/j.neuroimage.2006.06.009 16905335

[B25] Humphreys G. F. Lambon Ralph M. A. (2014). Fusion and fission of cognitive functions in the human parietal cortex. Cereb. Cortex [Epub ahead of print]. 10.1093/cercor/bhu198 25205661 PMC4585503

[B26] Jones D. K. Simmons A. Williams S. C. R. Horsfield M. A. (1999). Non-invasive assessment of axonal fiber connectivity in the human brain via diffusion tensor MRI. Magn. Reson. Med. 42, 37–41. 10.1002/(sici)1522-2594(199907)42:1<37::aid-mrm7>3.0.co;2-o 10398948

[B27] Jones D. K. Symms M. R. Cercignani M. Howard R. J. (2005). The effect of filter size on VBM analyses of DT-MRI data. Neuroimage 26, 546–554. 10.1016/j.neuroimage.2005.02.013 15907311

[B28] Kaufmann L. Wood G. Rubinsten O. Henik A. (2011). Meta-analyses of developmental FMRI studies investigating typical and atypical trajectories of number processing and calculation. Dev. Neuropsychol. 36, 763–787. 10.1080/87565641.2010.549884 21761997

[B30] Klein E. Moeller K. Glauche V. Weiller C. Willmes K. (2013a). Processing pathways in mental arithmetic—Evidence from probabilistic fiber tracking. PLoS One 8:e55455. 10.1371/journal.pone.0055455 23383194 PMC3559478

[B31] Klein E. Moeller K. Willmes K. (2013b). A neural disconnection hypothesis on impaired numerical processing. Front. Hum. Neurosci. 7:663. 10.3389/fnhum.2013.00663 24155704 PMC3805030

[B32] Klein E. Suchan J. Moeller K. Karnath H.-O. Knops A. Wood G.. (2014). Considering structural connectivity in the triple code model of numerical cognition: differential connectivity for magnitude processing and arithmetic facts. Brain Struct. Funct. [Epub ahead of print]. 10.1007/s00429-014-0951-1 25432772

[B85] Klein E. Willmes K. Dressel K. Domahs F. Wood G. Nuerk H.-C.. (2010). Categorical and continuous–disentangling the neural correlates of the carry effect in multi-digit addition. Behav. Brain Funct. 6:70. 10.1186/1744-9081-6-70 21092129 PMC3002291

[B33] Krueger F. Landgraf S. van der Meer E. Deshpande G. Hu X. (2011). Effective connectivity of the multiplication network: a functional MRI and multivariate granger causality mapping study. Hum. Brain Mapp. 32, 1419–1431. 10.1002/hbm.21119 20715080 PMC6870371

[B34] Kucian K. Ashkenazi S. S. Hänggi J. Rotzer S. Jäncke L. Martin E.. (2014). Developmental dyscalculia: a dysconnection syndrome?. Brain Struct. Funct. 219, 1721–1733. 10.1007/s00429-013-0597-4 23783231

[B36] Lebel C. Rasmussen C. Wyper K. Andrew G. Beaulieu C. (2010). Brain microstructure is related to math ability in children with fetal alcohol spectrum disorder. Alcohol Clin. Exp. Res. 34, 354–363. 10.1111/j.1530-0277.2009.01097.x 19930234

[B35] Le Bihan D. Breton E. (1985). Imagerie de diffusion *in vivo* par resonance magnetique nucleaire. C. R. L’Académie Sci. 301, 1109–1112.

[B37] Li Y. Hu Y. Wang Y. Weng J. Chen F. (2013a). Individual structural differences in left inferior parietal area are associated with schoolchildren’s arithmetic scores. Front. Hum. Neurosci. 7:844. 10.3389/fnhum.2013.00844 24367320 PMC3854708

[B38] Li Y. Wang Y. Hu Y. Liang Y. Chen F. (2013b). Structural changes in left fusiform areas and associated fiber connections in children with abacus training: evidence from morphometry and tractography. Front. Hum. Neurosci. 7:335. 10.3389/fnhum.2013.00335 23847506 PMC3701285

[B86] López-Barroso D. Catani M. Ripollés P. Dell’Acqua F. Rodríguez-Fornells A. de Diego-Balaguer R. (2013). Word learning is mediated by the left arcuate fasciculus. Proc. Natl. Acad. Sci. U S A 110, 13168–13173. 10.1073/pnas.1301696110 23884655 PMC3740909

[B39] Lotze M. Beutling W. Loibl M. Domin M. Platz T. Schminke U.. (2011). Contralesional motor cortex activation depends on ipsilesional corticospinal tract integrity in well-recovered subcortical stroke patients. Neurorehabil. Neural Repair 26, 594–603. 10.1177/1545968311427706 22140195

[B40] Matejko A. (2014). White matter counts: brain connections help us do 2+2. Front. Young Minds 2:19 10.3389/frym.2014.00019

[B42] Matejko A. Ansari D. (2015). Drawing connections between white matter and numerical and mathematical cognition: a literature review. Neurosci. Biobehav. Rev. 48C, 35–52. 10.1016/j.neubiorev.2014.11.006 25446952

[B41] Matejko A. A. Price G. R. Mazzocco M. M. M. Ansari D. E. (2013). Individual differences in left parietal white matter predict math scores on the preliminary scholastic aptitude test. Neuroimage 66, 604–610. 10.1016/j.neuroimage.2012.10.045 23108272

[B43] Menon V. (2013). Developmental pathways to functional brain networks: emerging principles. Trends Cogn. Sci. 17, 627–640. 10.1016/j.tics.2013.09.015 24183779

[B44] Menon V. (in press). “Arithmetic in child and adult brain,” in Handbook of Mathematical Cognition, eds Cohen Kadosh R. Dowker A. (Oxford: Oxford University Press).

[B87] Molko N. Cachia A. Riviere D. Mangin J. F. Bruandet M. Le Bihan D.. (2004). Brain anatomy in Turner syndrome: evidence for impaired social and spatial-numerical networks. Cereb. Cortex 14, 840–850. 10.1093/cercor/bhh042 15054057

[B45] Mori S. Crain B. J. Chacko V. P. van Zijl P. C. (1999). Three-dimensional tracking of axonal projections in the brain by magnetic resonance imaging. Ann. Neurol. 45, 265–269. 10.1002/1531-8249(199902)45:2<265::aid-ana21>3.0.co;2-3 9989633

[B46] Mukherjee P. Hess C. P. Xu D. Han E. T. Kelley D. A. Vigneron D. B. (2008). Development and initial evaluation of 7-T q-ball imaging of the human brain. Magn. Reson. Imaging 26, 171–180. 10.1016/j.mri.2007.05.011 17692489 PMC2997615

[B47] Navas-Sánchez F. J. Alemán-Gómez Y. Sánchez-Gonzalez J. Guzmán-De-Villoria J. A. Franco C. Robles O.. (2014). White matter microstructure correlates of mathematical giftedness and intelligence quotient. Hum. Brain Mapp. 35, 2619–2631. 10.1002/hbm.22355 24038774 PMC6868969

[B48] Newton J. M. Ward N. S. Parker G. J. Deichmann R. Alexander D. C. Friston K. J.. (2006). Non-invasive mapping of corticofugal fibres from multiple motor areas–relevance to stroke recovery. Brain 129, 1844–1858. 10.1093/brain/awl106 16702192 PMC3718077

[B49] Park J. Li R. Brannon E. M. (2014). Neural connectivity patterns underlying symbolic number processing indicate mathematical achievement in children. Dev. Sci. 17, 187–202. 10.1111/desc.12114 24267664

[B50] Park J. Park D. C. Polk T. A. (2013). Parietal functional connectivity in numerical cognition. Cereb. Cortex 23, 2127–2135. 10.1093/cercor/bhs193 22784605 PMC3729197

[B51] Peters B. D. Szeszko P. R. Radua J. Ikuta T. Gruner P. DeRosse P.. (2012). White matter development in adolescence: diffusion tensor imaging and meta-analytic results. Schizophr. Bull. 38, 1308–1317. 10.1093/schbul/sbs054 22499780 PMC3494037

[B88] Pickering S. Gathercole S. E. (2001). Working Memory Test Battery for Children (WMTB-C). UK: Psychological Corporation.

[B52] Poupon C. Clark C. A. Frouin V. Régis J. Bloch I. Le Bihan D.. (2000). Regularization of diffusion-based direction maps for the tracking of brain white matter fasciculi. Neuroimage 12, 184–195. 10.1006/nimg.2000.0607 10913324

[B53] Qin S. Cho S. Chen T. Rosenberg-Lee M. Geary D. C. Menon V. (2014). Hippocampal-neocortical functional reorganization underlies children’s cognitive development. Nat. Neurosci. 17, 1263–1269. 10.1038/nn.3788 25129076 PMC4286364

[B54] Rauschecker J. P. Scott S. K. (2009). Maps and streams in the auditory cortex: nonhuman primates illuminate human speech processing. Nat. Neurosci. 12, 718–724. 10.1038/nn.2331 19471271 PMC2846110

[B55] Reinvang I. (1985). Aphasia and Brain Organization. Berlin: Springer.

[B56] Rijntjes M. Weiller C. Bormann T. Musso M. (2012). The dual loop model: its relation to language and other modalities. Front. Evol. Neurosci. 4:9. 10.3389/fnevo.2012.00009 22783188 PMC3388276

[B58] Rosenberg-Lee M. Ashkenazi S. Chen T. Young C. B. Geary D. C. Menon V. (2015). Brain hyper-connectivity and operation-specific deficits during arithmetic problem solving in children with developmental dyscalculia. Dev. Sci. 18, 351–372. 10.1111/desc.12216 25098903 PMC4320038

[B59] Rosenberg-Lee M. Barth M. Menon V. (2011). What difference does a year of schooling make? Maturation of brain response and connectivity between 2nd and 3rd grades during arithmetic problem solving. Neuroimage 57, 796–808. 10.1016/j.neuroimage.2011.05.013 21620984 PMC3165021

[B60] Rusconi E. Pinel P. Eger E. LeBihan D. Thirion B. Dehaene S.. (2009). A disconnection account of Gerstmann syndrome: functional neuroanatomy evidence. Ann. Neurol. 66, 654–662. 10.1002/ana.21776 19938150

[B61] Rykhlevskaia E. Uddin L. Q. Kondos L. Menon V. (2009). Neuroanatomical correlates of developmental dyscalculia: combined evidence from morphometry and tractography. Front. Hum. Neurosci. 3:51. 10.3389/neuro.09.051.2009 20046827 PMC2796911

[B62] Sagi Y. Tavor I. Hofstetter S. Tzur-Moryosef S. Blumenfeld-Katzir T. Assaf Y. (2012). Learning in the fast lane: new insights into neuroplasticity. Neuron 73, 1195–1203. 10.1016/j.neuron.2012.01.025 22445346

[B63] Saur D. Kreher B. W. Schnell S. Kuemmerer D. Kellermeyer P. Vry M.-S.. (2008). Ventral and dorsal pathways for language. Proc. Natl. Acad. Sci. U S A 105, 18035–18040. 10.1073/pnas.0805234105 19004769 PMC2584675

[B64] Schlaug G. Marchina S. Norton A. (2009). Evidence for plasticity in white-matter tracts of patients with chronic Broca’s aphasia undergoing intense intonation-based speech therapy. Ann. N Y Acad. Sci. 1169, 385–394. 10.1111/j.1749-6632.2009.04587.x 19673813 PMC2777670

[B89] Simon O. Kherif F. Flandin G. Poline J. B. Rivière D. Mangin J. F.. (2004). Automatized clustering and functional geometry of human parietofrontal networks for language, space and number. Neuroimage 23, 1192–1202. 10.1016/j.neuroimage.2004.09.023 15528119

[B90] Simon O. Mangin J. F. Cohen L. Le Bihan D. Dehaene S. (2002). Topographical layout of hand, eye, calculation and language-related areas in the human parietal lobe. Neuron 33, 475–487. 10.1016/S0896-6273(02)00575-5 11832233

[B66] Soman S. Holdsworth S. J. Skare S. Andre J. B. Van A. T. Aksoy M.. (2015). Effect of number of acquisitions in diffusion tensor imaging of the pediatric brain: optimizing scan time and diagnostic experience. J. Neuroimaging 25, 296–302. 10.1111/jon.12093 24593174

[B67] Supekar K. Swigart A. G. Tenison C. Jolles D. D. Rosenberg-Lee M. Fuchs L.. (2013). Neural predictors of individual differences in response to math tutoring in primary-grade school children. Proc. Natl. Acad. Sci. U S A 110, 8230–8235. 10.1073/pnas.1222154110 23630286 PMC3657798

[B91] Tang Y. Zhang W. Chen K. Feng S. Ji Y. Shen J.. (2006). Arithmetic processing in the brain shaped by cultures. Proc. Natl. Acad. Sci. U S A 103, 10775–10780. 10.1073/pnas.0604416103 16815966 PMC1502307

[B82] Thiebaut de Schotten M. T. Tomaiuolo F. Aiello M. Merola S. Silvetti M. Lecce F.. (2014). Damage to white matter pathways in subacute and chronic spatial neglect: a group study and 2 single-case studies with complete virtual “in vivo” tractography dissection. Cereb. Cortex 24, 691–706. 10.1093/cercor/bhs351 23162045

[B68] Till C. Deotto A. Tipu V. Sled J. G. Bethune A. Narayanan S.. (2011). White matter integrity and math performance in pediatric multiple sclerosis: a diffusion tensor imaging study. Neuroreport 22, 1005–1009. 10.1097/wnr.0b013e32834dc301 22045260

[B69] Tsang J. M. Dougherty R.-F. Deutsch G. K. Wandell B. A. Ben-Shachar M. (2009). Frontoparietal white matter diffusion properties predict mental arithmetic skills in children. Proc. Natl. Acad. Sci. U S A 106, 22546–22551. 10.1073/pnas.0906094106 19948963 PMC2799736

[B70] Umarova R. M. Saur D. Schnell S. Kaller C. P. Vry M. S. Glauche V.. (2010). Structural connectivity for visuospatial attention: significance of ventral pathways. Cereb. Cortex 20, 121–129. 10.1093/cercor/bhp086 19406904

[B71] Van Beek L. Ghesquière P. Lagae L. De Smedt B. (2014). Left fronto-parietalwhite matter correlates with individual differences in children’s ability to solve additions and multiplications: a tractography study. Neuroimage 90, 117–127. 10.1016/j.neuroimage.2013.12.030 24368261

[B72] van Eimeren L. Grabner R. H. Koschutnig K. Reishofer G. Ebner F. Ansari D. (2010). Structure-function relationships underlying calculation: a combined diffusion tensor imaging and fMRI study. Neuroimage 52, 358–363. 10.1016/j.neuroimage.2010.04.001 20382234

[B73] van Eimeren L. Niogi S. N. McCandliss B. D. Holloway I. D. Ansari D. (2008). White matter microstructures underlying mathematical abilities in children. Neuroreport 19, 1117–1121. 10.1097/wnr.0b013e328307f5c1 18596611

[B92] von Aster M. Weinhold Zulauf M. Horn R. R. (2005). ZAREKI-R. Testverfahren zur Dyskalkulie bei Kindern. Göttingen: Hogrefe.

[B93] Wechsler D. (1999). Wechsler Abbreviated Scale of Intelligence. New York, NY: The Psychological Corporation: Harcourt Brace and Company.

[B94] Wechsler D. (2004). The Wechsler Intelligence Scale for Children—Fourth Edition. London: Pearson Assessment.

[B95] Wechsler D. (2005). Wechsler Individual Achievement Test 2nd Edition (WIAT II). London: The Psychological Corporation.

[B74] Weiller C. Bormann T. Saur D. Musso M. Rijntjes M. (2011). How the ventral pathway got lost: and what its recovery might mean. Brain Lang. 118, 29–39. 10.1016/j.bandl.2011.01.005 21429571

[B75] Wernicke C. (1874). Der Aphasische Sysmtomenkomplex. Breslau: Cohn and Weingart.

[B76] Wilkinson G. S. Robertson G. J. (2006). WRAT-4 Wide Range Achievement Test Professional Manual. Lutz: Psychological Assessment Resources, Inc.

[B77] Willmes K. Moeller K. Klein E. (2014). Where numbers meet words: a common ventral network for semantic classification. Scand. J. Psychol. 55, 202–211. 10.1111/sjop.12098 24605865

